# A Synergy Cropland of China by Fusing Multiple Existing Maps and Statistics

**DOI:** 10.3390/s17071613

**Published:** 2017-07-12

**Authors:** Miao Lu, Wenbin Wu, Liangzhi You, Di Chen, Li Zhang, Peng Yang, Huajun Tang

**Affiliations:** 1 Key Laboratory of Agricultural Remote Sensing, Ministry of Agriculture/Institute of Agricultural Resources and Regional Planning, Chinese Academy of Agricultural Sciences, Beijing 100081, China; lumiao@caas.cn (M.L.); l.you@cgiar.org (L.Y.); chendicaas@163.com (D.C.); zhangli05@caas.cn (L.Z.); yangpeng@caas.cn (P.Y.); tanghuajun@caas.cn (H.T.); 2 International Food Policy Research Institute, Washington, DC 20006, USA

**Keywords:** synergy map, cropland mapping, data fusion, statistics, agreement

## Abstract

Accurate information on cropland extent is critical for scientific research and resource management. Several cropland products from remotely sensed datasets are available. Nevertheless, significant inconsistency exists among these products and the cropland areas estimated from these products differ considerably from statistics. In this study, we propose a hierarchical optimization synergy approach (HOSA) to develop a hybrid cropland map of China, circa 2010, by fusing five existing cropland products, i.e., GlobeLand30, Climate Change Initiative Land Cover (CCI-LC), GlobCover 2009, MODIS Collection 5 (MODIS C5), and MODIS Cropland, and sub-national statistics of cropland area. HOSA simplifies the widely used method of score assignment into two steps, including determination of optimal agreement level and identification of the best product combination. The accuracy assessment indicates that the synergy map has higher accuracy of spatial locations and better consistency with statistics than the five existing datasets individually. This suggests that the synergy approach can improve the accuracy of cropland mapping and enhance consistency with statistics.

## 1. Introduction

Knowledge of the extent and dynamics of cropland is important for food security and environmental sustainability. Estimates of the global population in 2050 exceed 9 billion, with the resulting growing demand for food [1,2]. Changes in dietary patterns with rising income will increase demands for animal protein, vegetable oils, fruits, and vegetables, which implies that the cropland needed to support average human diets will increase [3]. These changes will undoubtedly put greater pressure on already overstretched cropland. Moreover, farming activities have enormous environmental consequences because they alter a large proportion of structure and function of ecosystems, which influences the ecosystem interaction with the surrounding atmosphere, aquatic systems and soils [4,5]. Thus, one of the greatest human challenges is to ensure that agricultural land systems can supply our society with sufficient, safe, and nutritious foods while reducing their negative impacts on the environment. Meeting these goals requires accurate information on cropland distribution and its spatiotemporal changes to support agricultural monitoring, food production estimation, food security assessment, and geophysical models [6,7].

Satellite images provide an efficient data source from which to derive cropland extent over large areas. In recent decades, several global and continental cropland datasets have been derived from remotely sensed data and made freely available to the public. Early cropland products generally employed coarse resolution images (pixels of 1 km or similar), such as International Geosphere-Biosphere Programme Data and Information Systems Cover (IGBP-DISCover) product [8], University of Maryland (UMD) product [9], and Global Land Cover 2000 (GLC2000) dataset [10]; however low accuracy and image quality limited their application at national or regional levels. Later, the spatial resolution of cropland datasets was improved to 500 m and then 300 m, such as MODIS Collection 5 (MODIS C5) Land Cover Products, and GlobCover 2009 [11,12]. Today, land cover mapping with high-resolution images has gained increasing attention in the scientific community because of the free availability of the Landsat archive, which has allowed production of 30 m resolution land cover datasets such as GlobeLand30 and Finer Resolution Observation and Monitoring-Global Land Cover (FROM-GLC) [13,14]. However, there is considerable inconsistency among these cropland mapping products, especially in heterogeneous landscape areas such as transition zones, as they have been produced using data from different sensors, using different classification features and classifiers [15,16]. Some studies have suggested that discrepancies also occur as a result of different classification schemes and cropland definitions [17,18,19]. Furthermore, the cropland areas estimated by these products differ from the official statistics, because mixed pixels are inevitable in remote sensing images, especially in coarse images [20,21]. Although some maps, such as GlobCover 2009 and Climate Change Initiative Land Cover (CCI-LC), contain mosaic cropland classes representing a combination of multiple classes, it is quite difficult to estimate cropland areas from maps with high accuracy because they do not provide precise percentages of the cropland [18].

The synergy approach, by fusing available datasets and statistics, provides an alternative to solve the above issues [22]. Different cropland products can be used to generate a hybrid percentage map of cropland, often with improved accuracy. Existing algorithms for map synergy can be broadly categorized into regression methods and agreement scoring methods. In the former, geographically weighted regression (GWR) is a spatial analytical method with regression parameters to vary over space [23]. It has been widely used to build hybrid land cover and forest maps, at global scale, by using crowdsourced data from Geo-wiki [24,25]. However, this process generally requires large training sample sets and tends to be unstable when sampling density is low [26,27]. The agreement scoring method, with score assignment based on levels of agreement in the input data, is more widely used because of its simplicity and superior operability. Using this method, Jung et al., developed a global land cover map of 1 km resolution for carbon cycle modeling [28], and Ramankutty et al., developed global cropland and pasture extent maps [29]. However, these studies ignored the quality differences between the input datasets. To address this problem, Fritz et al., used expert knowledge to rank the input datasets and assign different weights accordingly [30,31]. It is well recognized that score assignment is the key component of these methods. For a given number n of input data, there are 2^n^ combinations in the scoring table. Setting up the scoring table can be strenuous and time-consuming when input datasets are numerous.

This study improves and simplifies the above method by developing a new hierarchical optimization synergy approach (HOSA) based on agreement of input cropland products. The approach includes determination of the optimal agreement level and identification of the best combination of datasets. HOSA was tested by fusing five cropland datasets (GlobeLand30, CCI-LC, GlobCover 2009, MODIS C5, and MODIS Cropland) and statistics of cropland area to create a synergic cropland map of China, circa 2010.

## 2. Data and Method

### 2.1. Data Sources

The main inputs to this study were remote sensing-derived global cropland datasets, cropland sampling points, and cropland area statistics. Five cropland datasets, i.e., GlobeLand30, CCI-LC, GlobCover 2009, MODIS C5 and MODIS Cropland, developed through different national or international initiatives, were selected to create a synergic cropland map of China for the year of circa 2010. There are some significant differences between them in terms of spatial resolution, data sources, time coverage, methods and accuracies (Table 1 and Table 2). GlobeLand30 was produced using the 30 m spatial resolution images from Landsat and China HJ-1, and generated by a pixel-object-knowledge (POK)-based classification approach [13]. CCI-LC was generated by the Land Cover project of the Climate Change Initiative led by the European Space Agency (ESA). The MERIS (Medium Resolution Imaging Spectrometer Instrument) time series data from 2008 to 2012 were used to develop the CCI-LC maps at 300 m spatial resolution. Built on the unsupervised classification chain, CCI-LC added machine learning classification to improve the accuracy [32]. GlobCover 2009 was implemented by the ESA and the Université Catholique de Louvain (UCL), and was primarily based on unsupervised and supervised clustering of MERIS Fine Resolution (FR) time series data at a spatial resolution of 300 m [12]. MODIS C5 was produced by Boston University with 500 m spatial resolution. A decision tree was implemented using the spectral and temporal features of MODIS bands 1–7 and the enhanced vegetation index, with Bayesian rules used to adjust the class-conditional probabilities [11]. The MODIS Cropland extent map was based on 250 m MODIS data from 2000 to 2008. Using the multiyear MODIS metrics, a decision tree was applied to calculate the probability of cropland. Then, thresholds were set to determine the cropland extent according to the cropland area statistics [33]. Although there are slight differences in temporal coverage among these cropland maps (Table 1), they has a minor impact on the final fusing map in 2010 as the inconsistency among the input datasets is farther larger than the time difference.

**Figure 1:**
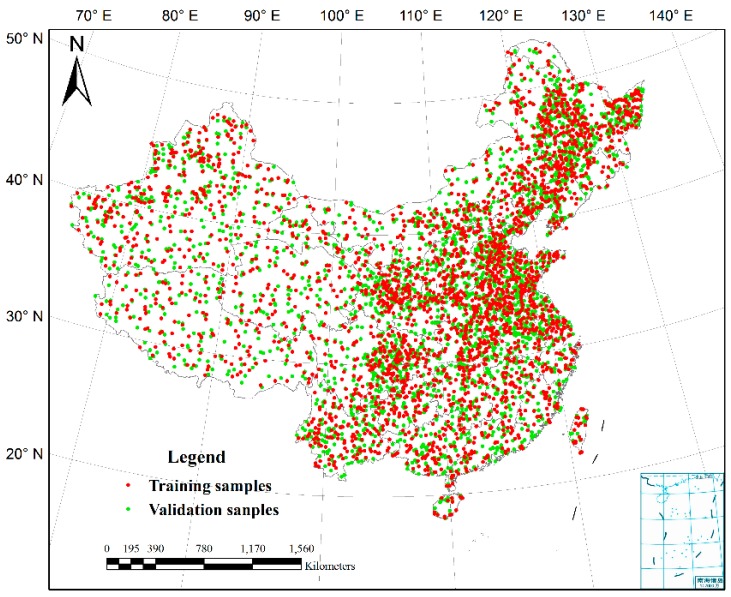
Training and validation samples.

Cropland sampling points were used to assess the accuracy of existing cropland datasets and for validation of the synergic cropland map, which were provided by Tsinghua University [14]. In the sampling frame of Tsinghua University, entire globe was divided by hexagonal scheme using DGGRID software, and then 10 samples were assigned randomly in each hexagon. The land cover types of sampling points were identified by visual interpretation via high resolution images. Although this sampling method can ensure the uniformity and objectivity of the distribution of the samples, the total number of cropland samples in China was only 443, which is insufficient for evaluation. Thus, a stratified random sampling method based on agreement of the five cropland mapping products was used to collect more samples, with land cover types determined by visual interpretation of Google Earth images. The images in 2010 or around 2010 were generally used for sample selection. When the images around 2010 were not available, time slider of Google Earth was moved to search for the images before 2010 and after 2010. These images were compared to determine the land cover types of the samples. If the sample remains unchange before and after 2010, it was considered to be a sampling point, otherwise it was removed. The final numbers of cropland and noncropland samples were 2800 and 2851, respectively. Approximately half of the total sampling points were used for training to rank the five cropland maps, and the rest were used for validation to assess the accuracy of the new hybrid cropland map (Figure 1). Statistics of cropland area at provincial level in the base year of 2009 were acquired from the Second National Land Survey. The cropland definition of statistics is similar to that used by the Food and Agriculture Organization (FAO) of the United Nations. These statistics were estimated using cropland polygons derived by visual interpretation of aerial photographs with ground survey assistance, and the areas exclude the cropland ridge.

### 2.2. Data Processing

Data preparation included transformation of coordinates, harmonization of cropland definitions, and standardization of the spatial resolution. First, all five cropland datasets were reprojected to geographic latitude/longitude coordinate system, with WGS84 datum, using nearest-neighbor resampling. Because these datasets vary in their classification schemes (Table 2), it was necessary to harmonize the cropland definition prior for data fusion.

The FAO definition, which includes arable land and permanent crops, was used as the standard. Pure cropland classes, generally with higher accuracies, were assigned higher percentage weights, while mosaic cropland classes with lower accuracies, were assigned lower weights followed the definition of the corresponding classification scheme. For instance, GlobCover 2009 contains four cropland classes: two pure and two mixed. The two pure cropland classes of “post-flooding or irrigated croplands” and “rained croplands” were deemed 100% cropland. For the mixed classes “mosaic cropland (50–70%)/vegetation (20–50%)” and “mosaic vegetation (50–70%)/cropland (20–50%)”, the percentages of cropland were assigned in accordance with the stated proportions. With these rules, the land cover products were converted to cropland maps at their native spatial resolution. After harmonization of cropland definitions, all these datasets were standardized to the spatial resolution of 0.41667° with average cropland percentage, which is nearly equal 500 m.

### 2.3. Methodology

The overall principle of HOSA is that a pixel with greater agreement among existing cropland datasets is more likely to truly be a cropland pixel. There are two steps in HOSA to generate the synergy cropland map. The first step is to determine the optimal agreement level. The second step is to identify the best combination of datasets. To evaluate the performance of this method, the synergy cropland map is compared with the input cropland datasets using the accuracies of spatial location and cropland area.

**Figure 2:**
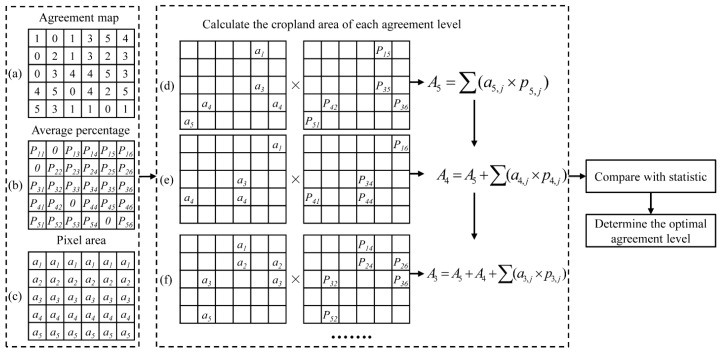
Flowchart on determination of the optimal agreement level. (**a**) Agreement map; (**b**) Average percentage; (**c**) Pixel area; (**d**–**f**) Calculations of cropland area with agreement level 5, 4, and 3 respectively.

(1) Determination of the optimal agreement level. Agreement is an important criterion for combining different land cover maps to create an improved dataset [31]. Because greater agreement between cropland maps indicates greater likelihood of true cropland, the optimal agreement level is determined by ranking cropland areas from high to low agreement and then allocating the highest ranked pixels as cropland until the statistical total area is reached. Figure 2 shows the flowchart for determining the optimal agreement level using the five existing products. First, overlay analysis of the five input datasets produces an agreement map and an average percentage map. The agreement map, with each pixel value ranging from 0 to 5, indicates the number of cropland products that label the pixel as cropland (Figure 2a). Then, in each pixel, the percentage of cropland is averaged to obtain the average percentage map (Figure 2b). For geographic latitude/longitude coordinate system, the area of each pixel becomes smaller from equator to the poles on the sphere. Therefore, the equal-area projection is used to calculate projected area for each pixel (Figure 2c). Second, the cropland area is summarized for the pixels ranking from high to low agreement level and compared with the statistics.

As shown in Figure 2d, the overall cropland area is aggregated by adding together the highest agreement pixel areas multiplied by the cropland percentage. If the area so aggregated is less than the statistical, cropland area of lower agreement is added (Figure 2e) until the area is equal to, or slightly greater than, the statistical area. In this way, the optimal agreement level is determined.

(2) Identification of the best product combination. The optimal agreement level indicates the number of cropland products involved in mapping, but does not indicate which products were used in the synergy. The product combination is identified by establishment of a score table in which products are ranked by accuracy. Using the training samples of Figure 1, the accuracies of the five products were assessed using an error matrix. The input products were ranked according to their overall accuracies. The product with the highest accuracy assessment was designated as product #1, followed by products #2, #3, #4, and #5. When the optimal agreement level is 3, there are 10 possible combinations (Table 3). A combination using higher-ranked products has a higher score. The cropland statistics are then used to identify the appropriate product combinations. First, the pixels with the highest score are selected, and their cropland area is calculated by the average cropland percentage multiplied by the corresponding pixel area. Pixels with the next highest score are then selected and added to the previous pixels. The new total area is then calculated. This step is repeated until the mapped cropland area matches the statistical area as closely as possible. The synergy cropland map is then created using the determined product combinations.

(3) Assessment of the synergy cropland map. The accuracies of the five input datasets and synergy map were evaluated and compared based on the validation samples (Figure 1) using error matrix [34,35]. One of the purposes of the synergy approach is to solve the inconsistency between cropland map and statistics. Therefore, the cropland areas of synergy map and the five cropland datasets were calculated in each province of China, and compared with statistics using the following quality indicators: correlation coefficient (*R*, Equation (1)), root mean square error (*RMSE*, Equation (2)), absolute difference (*AD*, Equation (3)), and average absolute relative difference (*AARD*, Equation (4)):

(1) $$R=\frac{\sum_{i=1}^{n}\left(a_{i}-\bar{a})(s_{i}-\bar{s}\right)}{\sqrt{\sum_{i=1}^{n}{\left(a_{i}-\bar{a}\right)}^{2}\cdot \sum_{i=1}^{n}{\left(s_{i}-\bar{s}\right)}^{2}}}$$

(2) $$RMSE=\sqrt{\frac{\sum_{i=1}^{n}\left(a_{i}-s_{i}\right)}{n}}$$

(3) $$AD=\frac{1}{n}\sum_{i=1}^{n}\left(a_{i}-s_{i}\right)$$

(4) $$AARD=\frac{1}{n}\sum_{i=1}^{n}\left|\left(a_{i}-s_{i}\right)/s_{i}\right|$$

where *a_i_* is the cropland area of province *i* estimated by cropland dataset or synergy map; *s_i_* is the statistical cropland area as the reference; $\bar{a}$ and $\bar{s}$ are the means of the estimated cropland areas and statistical cropland areas, respectively; and *n* is the number of provinces.

## 3. Results and Analysis

### 3.1. Agreement Analysis and Accuracy Assessment of the Five Cropland Datasets

**Figure 3:**
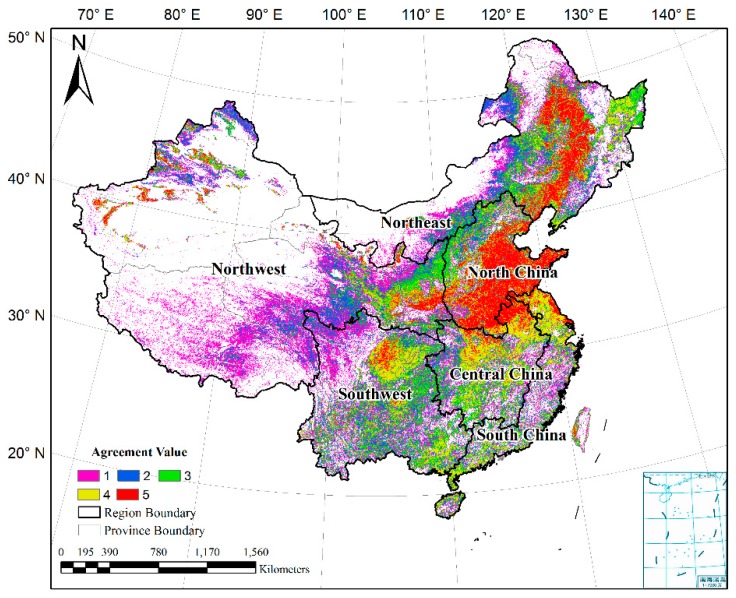
Spatial agreement of the five input datasets in the six regions of China.

**Figure 4:**
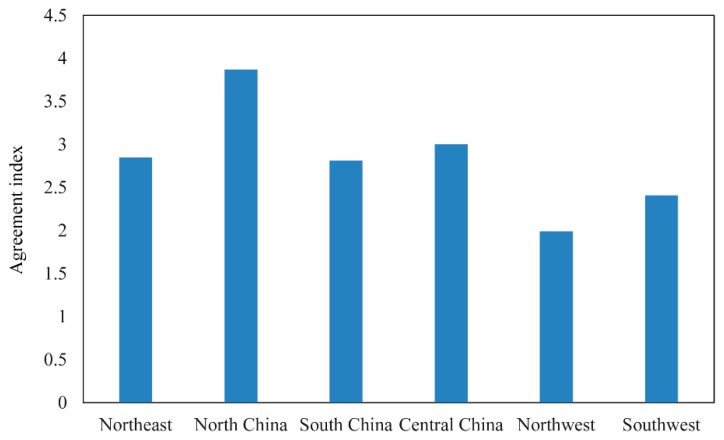
Value of the agreement index in each of the six regions.

Figure 3 shows the areas of agreement and disagreement of the five cropland datasets. The values 1–5 stand for the agreements for pixels. A higher value indicates higher agreement, i.e., a value of 5 reflects complete agreement between the five datasets; a value of 4 indicates that four datasets classify the pixel as cropland. In order to simplify analyses, the study area was divided into six regions according to the neighborhood provinces, i.e., the northeast, north, central, south, northwest, and southwest parts of China (Figure 3). High agreement is shown in the large homogeneous regions, such as the plains in northeast and north China, which are the major grain-producing areas. The spatial agreement becomes lower in moving from these major grain-producing regions to the farming-pastoral areas, and is lowest in the pastoral areas. The agreement index, the mean of the agreement values in each region, was used to determine the spatial agreement quantitatively (Figure 4). North China have the highest agreement index (3.87), followed by central and northeast China. As the major grain-producing regions, the cropland areas are intensively used and continuously distributed because of the homogeneous landscape. By comparison, the northwest has the lowest agreement index. Because this region is typified by farming-pastoral areas (such as Shaanxi and Ningxia provinces) and pastoral areas (such as Tibet and Xinjiang provinces), there is a wide mosaic of cropland and pasture, making classification difficult. Because the classification systems of CCI-LC and GlobCover 2009 include mosaic classes of cropland, low spatial agreements in the northwest of China are obvious.

**Figure 5:**
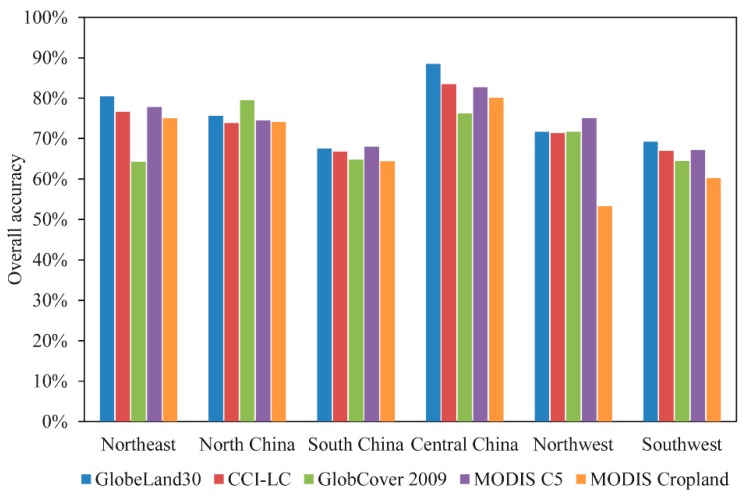
Accuracy assessment of the five input datasets in each region based on training samples.

The accuracies of the input datasets are crucial for building the score table. Figure 5 presents their overall accuracies assessed using an error matrix based on training samples in each region. In northeast, central, and southwest China, GlobeLand30 achieves the highest overall accuracies (80.50%, 88.47%, and 69.23%, respectively). In south and northwest China, MODIS C5 produces the highest accuracies (68.00% and 75.09%), and GlobCover 2009 achieves the best performance in north China with overall accuracy of 79.52%. Among the five datasets, MODIS Cropland has lower accuracies in most regions. Using the overall accuracies, the five input products were ranked in each province to build the corresponding score table.

### 3.2. Cropland Map Developed by HOSA

**Figure 6:**
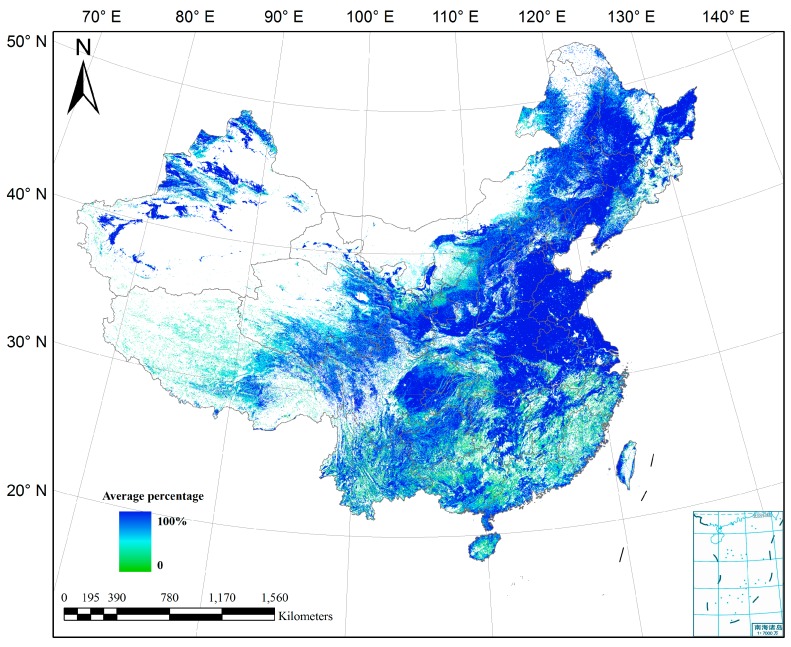
Average cropland percentage of the five input datasets.

**Figure 7:**
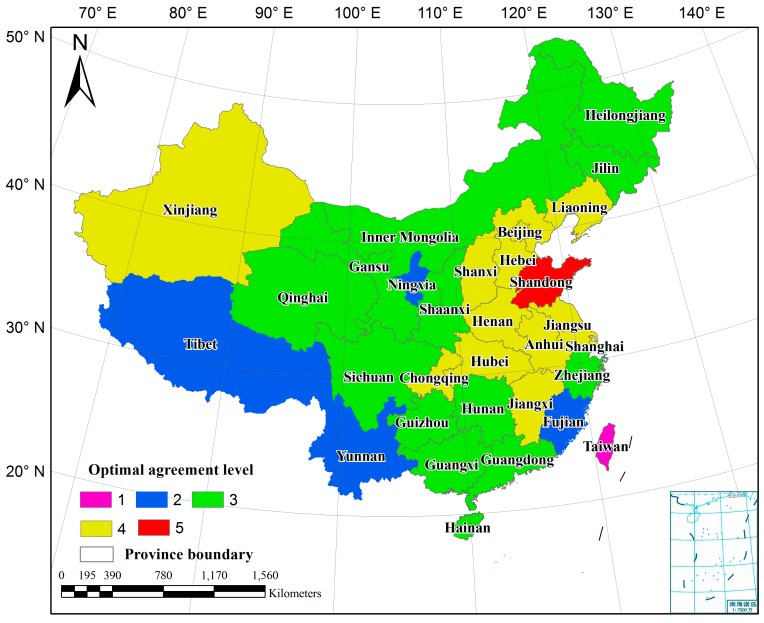
Optimal agreement level in each province.

The synergy processing was executed with each province as the operating unit. The average cropland percentage of the five input datasets was calculated. In Figure 6, blue areas indicate higher percentage and green lower percentage. In the homogeneous areas, such as plains and basins, the percentage is high, while heterogeneous areas have low average percentages, especially in the hills of south China and plateaus of the southeast and northeast regions. For each province, cropland area was summarized from high to low agreement level, and compared with the statistical area to determine the optimal agreement level. Figure 7 shows the optimal agreement level of each province. Overall, from eastern China to the west, the optimal agreement level gradually decreases. Specifically, the major grain-producing area of Shandong province has the highest agreement level of 5, which indicates than the sum cropland area of agreement 5 (Figure 3) can stratify the statistics. The optimal agreement level of 4 is seen in the provinces along the eastern coast and in the plains of central and northern China, such as Henan, Hubei, and Jiangsu. In some western provinces, the optimal agreement levels decrease to 3. Tibet, Yunnan, Ningxia, and Fujian provinces have optimal agreement levels of 2. Because of complicated topography and fragmented landscapes, mixed pixels are quite common in these regions, resulting in lower agreement between datasets. By comparison, the agreement level of Taiwan is 1, the lowest. The statistics report the cropland area of Taiwan as 1.2 million ha, and the total area of pixels labeled as cropland of the five input datasets can satisfy the statistics.

**Figure 8:**
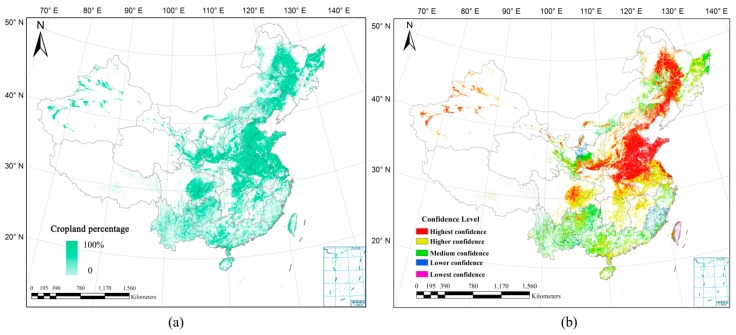
Synergy results based on the five cropland maps: (**a**) cropland percentage map; (**b**) cropland confidence map.

The overall accuracy of each cropland dataset was calculated for each province, and the cropland datasets were ranked using their overall accuracies. Next, a score table of product combination was established in each province, and the optimal combination of input datasets was determined using the statistical data for calibration. From the synergy results shown in Figure 8a, it is evident that high percentages of cropland are mainly located in the plains and basins (e.g., Weihe Plain, Huang-Huai-Hai Plain, Northeast Plain, Yangtze River Basin, and Sichuan Basin), while low percentages are found in plateaus and hills (e.g., Loess Plateau, Yunnan-Guizhou Plateau, Tibet Plateau, and Southeast Hills), which accords with the real situation. For each pixel of the cropland percentage map, a confidence level was defined according to the agreement value of the five input datasets. Because a high agreement value generally means high interpretation quality, the values from 1 to 5 were defined as lowest, lower, medium, higher and highest confidence, respectively, and the cropland confidence map can represent the quality of synergy result in detail (Figure 8b).

### 3.3. Accuracy Assessment

The accuracy of the synergy map was assessed by using an error matrix with the validation samples (Figure 1). The numbers of cropland and noncropland samples were 1422 and 1402, respectively. The validation results are shown in Table 4 with overall accuracy of 78.65% and Kappa coefficient of 0.57. The commission errors of cropland and noncropland are 21.87% and 20.83%, respectively; the omission errors of cropland and noncropland are 20.81% and 21.89%, respectively.

**Figure 9:**
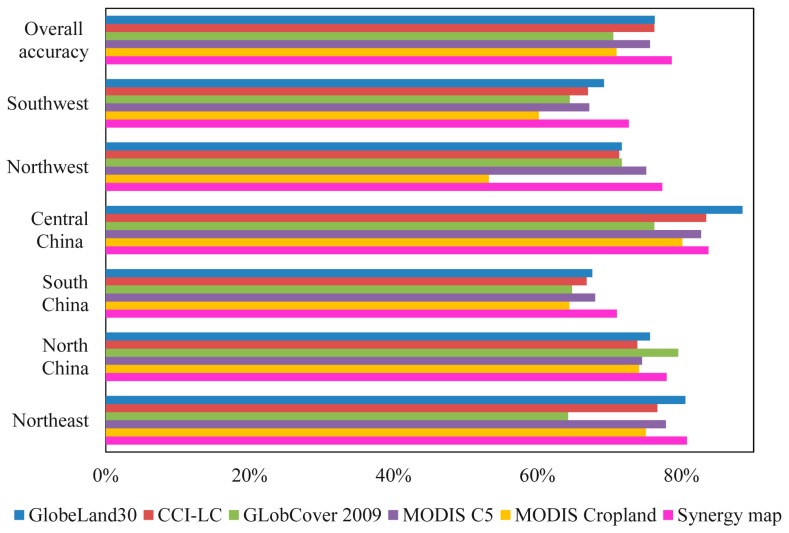
Overall and regional accuracies of the five cropland datasets and the synergy map.

The accuracy of synergy map was further compared with the original five cropland datasets. Error matrix was also used to assess the accuracies of the five cropland maps using the same test samples of synergy map. The overall accuracies of the input datasets and synergy result in the different regions are shown in Figure 9. The overall accuracy of the synergy map is higher than the input datasets. GlobeLand30 among the five cropland datasets has the highest overall accuracy (76.27%), followed by MODIS C5 (76.22%) and CCI-LC (74.22%). The accuracies of GlobCover 2009 and MODIS Cropland are relatively lower which are 70.50% and 70.96%, respectively. This conclusion agrees well with that by Yang et al., [36]. At the regional level, except for central China and north China, the synergy map has the highest overall accuracy in most regions. This implies that the synergy approach can take advantage of multiple datasets to establish a more accurate hybrid map.

### 3.4. Comparison with Statistics

**Figure 10:**
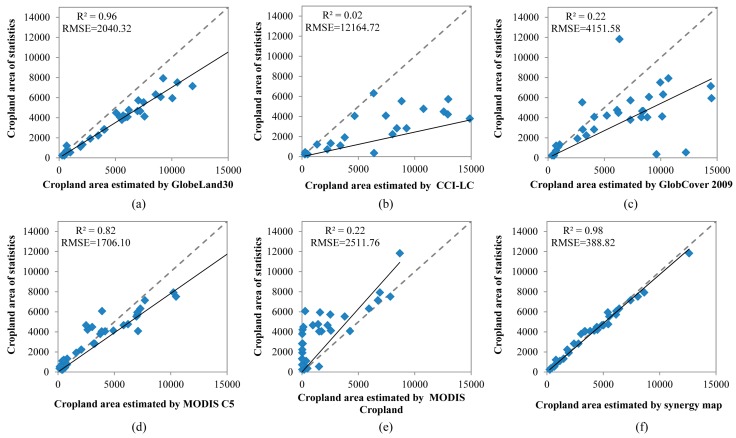
Scatterplots of cropland area from statistics and those estimated by GlobeLand30 (**a**); CCI-LC (**b**); GlobCover 2009 (**c**); MODIS C5 (**d**); MODIS Cropland (**e**) and synergy map (**f**).

The cropland areas of the input datasets and the synergic map were calculated and compared using quality indicators including *R*, *RMSE*, *AD*, and *AARD*. Figure 10 shows the relationships between cropland areas from statistics and estimated using the cropland datasets by province. In comparison with the five cropland datasets, we can see that the synergy map has the highest consistency with statistics, with a correlation coefficient of 0.98 and the lowest *RMSE* of 388.82. Among the input datasets, GlobeLand30 achieved a higher correlation coefficient (0.96) and lower *RMSE* (2040.32) than the other four datasets, which may be the result of its higher spatial resolution. The data points of CCI-LC (Figure 10b) deviate markedly from the 1:1 line and have the lowest correlation coefficient (0.02) and highest *RMSE* (12164.72), although its overall accuracy is higher (Figure 9). This may be caused by the definition of cropland including some mosaic types (Table 2).

The *AD* values describe the absolute differences of cropland area between cropland maps and statistics, and *AARD* values reflect the relative differences compared with statistics. As shown in Table 5, synergy map achieved the lowest *AD* and *AARD* values. The positive values of *AD* indicate that most of the datasets overestimated the cropland area, except MODIS Cropland, because of the mixed pixels in classification images and mosaic cropland definition [19]. Based on the analyses, it is clear that the synergy result has a better consistency with statistics. Therefore, the synergy approach can effectively solve the issue of inconsistency between cropland map and statistics.

## 4. Discussion

The mapping of cropland over large areas with high accuracy, especially in a cost-effective way, is always challenging. Here, we propose a new synergy method HOSA that aims to strike a balance between product accuracy and affordability. This approach is notable for its efficiency and accuracy of mapping using agreement between existing cropland datasets. We tested the method to produce a hybrid cropland map of China, circa 2010, with a spatial resolution of 500 m, using the five cropland datasets (GlobeLand30, CCI-LC, GlobCover 2009, MODIS C5 and MODIS Cropland), and sub-national statistics. Accuracy assessment showed that the synergy map had higher accuracy than these five input datasets, as well as better consistency with the cropland statistics. This confirms that the synergy approach can improve spatial accuracy and enhance consistency with statistics.

Compared with other synergy approaches, HOSA simplifies the method of score assignment [28,29,30,31]. Existing approaches built a long and static table to determine the cropland and noncropland areas, with 32 scores for the five input datasets, which was strenuous and time-consuming. Instead, HOSA first determined the optimal agreement level, and then built a dynamic score table in each province. The optimal agreement levels for most provinces ranged from 2 to 4 (Figure 7), and the score table was built according to the agreement level, which greatly reduced the size of the score table with an improved efficiency.

Because the statistical areas were obtained by visual interpretation from aerial photographs and ground survey, HOSA assumes that the statistics are the ‘true’ areas of cropland, and the statistics are therefore used in combination with the five cropland datasets to allocate equivalent areas within each province. The strength of HOSA is that it can combine such verified statistics from ground observation with the spatial detail of images [22]. However, the low spatial resolutions of the five input cropland datasets mean that mixed pixels are common, and there is a gap of spatial scale between the statistics and the cropland datasets. Remote sensing datasets often overestimate cropland area compared with statistics, because of mixed pixels and mosaic cropland classes [19,20]. As shown in Table 5, cropland areas estimated by GlobeLand 30, CCI-LC, GlobCover 2009 and MODIS C5 are higher that the statistics. To solve this problem, the percentage weights were applied for cropland datasets according to the definition of classification scheme (Table 2), and average percentages of cropland were calculated within the 500 m grids for resolution standardization. Finally, a cropland percentage map, unlike traditional cropland/noncropland maps, was generated and found to be consistent with the statistics. The way to determine the percentage weights of cropland classes is beyond the scope of this study, but it is worthy of being investigated in the future as it may influence the final map.

Building the score table is critical for identification of dataset combinations. Because cropland products generally have diverse accuracies in different regions, the score tables should be dynamic and region specific. Consequently, more samples are needed to assess accuracies in the corresponding regions. Although there are some platforms to collect samples by crowdsourcing methods, such as Geo-Wiki, Laco-Wiki, and Collect Earth [37,38,39], it is still a huge effort to collect numerous samples and assess their reliabilities. It is thus necessary to explore alternative methods of accuracy assessment that have less dependence on samples. In future work, we plan additional experiments in the accuraciy assessment of the input datasets using statistics as a reference.

The agreement between input cropland datasets is the basis of HOSA. Because higher agreement indicates more confidence in synergic results, the cropland confidence map (Figure 8b) can be treated as a spatially explicit indicator of map quality. It is clear that higher confidences appear in regions with homogeneous landscapes, while lower confidences appear in regions with heterogeneous landscapes. The limited accuracy and associated uncertainty of cropland datasets in heterogeneous areas restrict their application [4]. For these areas, the accuracy in a more informative way is needed to improve the quality of synergy result. GWR will be used to analyze spatial variation of accuracy and compared with confidence map [40,41]. Meanwhile, higher resolution and more accurate maps are needed for heterogeneous areas.

## 5. Conclusions

Accurate information on cropland spatial extent is important for a wide variety of applications. Although there are several cropland products, inconsistencies between them are large enough to restrict their application. HOSA is designed to produce a synergy cropland map cost-effectively, based on cropland datasets and statistics. This approach includes two optimization levels, i.e., determination of the optimal agreement level and determination of the best dataset combination, and can simplify the process of dataset synergy compared with traditional score assignment. HOSA was applied to generate a synergy cropland map of China, circa 2010, based on five cropland datasets and statistics. The results indicate that the synergy map has higher accuracy and better consistency with statistics than the original datasets. Therefore, the method can be extended further to regional or even global-scale cropland mapping. HOSA can also be used for other land cover classes, such as forest, grass, and water. In the future, more input products will be collected to improve the accuracy of synergy mapping, especially in regions with heterogeneous landscapes, and new method of accuracy assessment to reduce dependence on samples will be explored.

## Figures and Tables

**Table 1:** Characteristics of the five cropland datasets.

Dataset	Spatial Resolution	Sensor	Epoch	Classification Method
GlobeLand30	30 m	Landsat TM/HJ-1	2010	POK
CCI-LC	300 m	MERIS	2008–2012	Unsupervised/supervised clustering
GlobCover 2009	300 m	MERIS	2009	Unsupervised/supervised clustering
MODIS C5	500 m	MODIS	2010	Decision tree classification
MODIS Cropland	250 m	MODIS	2000–2008	Decision tree classification

**Table 2:** Cropland definition and percentage determination.

Dataset	Definition of Cropland	Cropland Accuracy Released by Producer	Cropland Percentage
GlobeLand30	Cultivated land	80.33%	100%
CCI-LC	Cropland, rainfed	85%	100%
	Herbaceous cover	__	80%
	Tree or shrub cover	__	80%
	Cropland, irrigated or post-flooding	88%	100%
	Mosaic cropland (>50%)/natural vegetation (tree, shrub, herbaceous cover) (<50%)	68%	60%
	Mosaic natural vegetation (tree, shrub, herbaceous cover) (>50%)/cropland (<50%)	63%	40%
GlobCover 2009	Post-flooding or irrigated croplands (or aquatic)	88%	100%
	Rainfed croplands	81%	100%
	Mosaic cropland (50–70%)/vegetation (20–50%)	64%	60%
	Mosaic vegetation (50–70%)/cropland (20–50%)	46%	40%
MODIS C5	Cropland	83.3%	100%
	Cropland/natural vegetation mosaics	60.5%	60%
MODIS Cropland	Cropland	__	100%

**Table 3:** Score table of product combinations when optimal agreement level is 3.

Score	#1	#2	#3	#4	#5
10	1	1	1	0	0
9	1	1	0	1	0
8	1	0	1	1	0
7	0	1	1	1	0
6	1	1	0	0	1
5	1	0	1	0	1
4	0	1	1	0	1
3	1	0	0	1	1
2	0	1	0	1	1
1	0	0	1	1	1

**Table 4:** Validation results of the synergy map.

		Validation Samples			
		Cropland	Noncropland	Sum	Commission Error
Synergy map	Cropland	1111	311	1422	21.87%
	Noncropland	292	1110	1402	20.83%
	Sum	1403	1421	2824	
	Omission error	20.81%	21.89%		
Overall accuracy = 78.65%, Kappa coefficient = 0.57					

**Table 5:** Comparison of *AD* and *AARD* values between cropland areas from statistics and estimated by the five datasets and synergy map.

	GlobeLand30	CCI-LC	GlobCover 2009	MODIS C5	MODIS Cropland	Synergy Map
*AD* (ha)	1585.11	8342.77	2357.00	499.88	–1895.28	12.02
*AARD*	0.45	3.50	2.00	0.32	0.65	0.09
